# Variation in rehabilitation setting after uncomplicated total knee or hip arthroplasty: a call for evidence-based guidelines

**DOI:** 10.1186/s12891-019-2570-8

**Published:** 2019-05-15

**Authors:** Justine M. Naylor, Andrew Hart, Ian A. Harris, Adriane M. Lewin

**Affiliations:** 1 South West Sydney Local Health District, Locked Bag 7103, Liverpool, NSW BC 1871 Australia; 2 South West Sydney Clinical School, UNSW, Liverpool, Australia; 3 Ingham Institute Applied Medical Research, Liverpool, Australia

**Keywords:** Arthroplasty, knee, Arthroplasty, hip: rehabilitation, Physical therapy

## Abstract

**Background:**

High-level evidence consistently indicates that resource-intensive facility-based rehabilitation does not provide better recovery compared to home programs for uncomplicated knee or hip arthroplasty patients and, therefore, could be reserved for those most impaired. This study aimed to determine if rehabilitation setting aligns with evidence regardless of insurance status.

**Methods:**

Sub-study within a national, prospective study involving 19 Australian high-volume public and private arthroplasty centres. Individuals undergoing primary arthroplasty for osteoarthritis participated. The main outcome was the proportion participating in each rehabilitation setting, obtained via chart review and participant telephone follow-up at 35 and 90 days post-surgery, categorised as ‘facility-based’ (inpatient rehabilitation and/or ≥ four outpatient-based sessions, including day-hospital) or ‘home-based’ (domiciliary, monitored or unmonitored home program only). We compared characteristics of the study cohort and rehabilitation setting by insurance status (public or private) using parametric and non-parametric tests, analysing the knee and hip cohorts separately.

**Results:**

After excluding ineligible participants (bilateral surgeries, self-funded insurance, participation in a concurrent rehabilitation trial, experience of a major acute complication potentially affecting their rehabilitation pathway), 1334 eligible participants remained. Complete data were available for 1302 (97%) [Knee: *n* = 610, mean age 68.7 (8.5) yr., 51.1% female; Hip: *n* = 692, mean age 65.5 (10.4) yr., 48.9% female]; 26% (158/610) of knee and 61% (423/692) of hip participants participated predominantly in home-based programs. A greater proportion of public recipients were obese and had greater pre-operative joint impairment, but participated more commonly in home programs [(Knee: 32.9% (79/240) vs 21.4% (79/370) (*P* = 0.001); Hip: 71.0% (176/248) vs 55.6% (247/444) (*P* <  0.001)], less commonly in inpatient rehabilitation [Knee: 7.5% (18/240) vs 56.0% (207/370) P (< 0.001); Hip: 4.4% (11/248) vs 33.1% (147/444) (*P* <  0.001], and had fewer outpatient treatments [Knee: median (IQR) 6 (3) vs 8 (6) (*P* < 0.001); Hip: 6 (4) vs 8 (6) (*P* < 0.001)].

**Conclusions:**

Facility-based programs remain the norm for most knee and many hip arthroplasty recipients with insurance status being a major determinant of care. Development and implementation of evidence-based guidelines may help resolve the evidence-practice gap, addressing unwarranted practice variation across the insurance sectors.

**Electronic supplementary material:**

The online version of this article (10.1186/s12891-019-2570-8) contains supplementary material, which is available to authorized users.

## Background

The lifelong risks for total knee arthroplasty (TKA) and total hip arthroplasty (THA) are increasing in Australia and elswhere [1, 2]. In Australia, approximately one in five women and one in seven men will have a TKA [1], and one in eight women and one in ten men will have a THA [2]. The increasing risks are largely attributed to the aging population [1, 3–5], and increasing obesity [1, 4, 6]. In 2017, 91,857 primary TKA and THA procedures were undertaken in Australia; most (TKA 70%; THA 67%) occurred in the private sector [7].

The patient-reported joint-specific improvements following TKA or THA are typically large [8–13], and evident within 3–12 months post-surgery. Further, the more generic health-related quality of life scores often reach the same level 1 year post-arthroplasty as those of the general population for the same age groups [9, 11–13]. In contrast, whilst measured physical performance such as gait speed and strength improves with either surgery, it remains well short of that of healthy age-matched persons [14–19].

Facility-based supervised physical therapy-based rehabilitation commencing shortly after discharge from acute care has become the accepted standard of care following TKA or THA [20–25], perhaps because of the shortfall in physical recovery in particular. Supervised therapy takes several forms, varying in the level of involvement and oversight of the physical therapist [22–27]. These include inpatient-based rehabilitation in a dedicated rehabilitation unit, outpatient (clinic-based) programs involving one-to-one or group-based therapy (on land or in water), day hospital visits, domiciliary care with therapists treating patients in client homes, or simple monitored programs including telerehabilitation where patients perform therapist-assigned exercises at home, with occasional oversight by a therapist.

Whilst a recent systematic review concluded that some form of physical therapy program is better than a minimal program (exercise instruction) or nothing following TKA [28], there is consistent evidence from randomized trials and a recent systematic review [29] that the setting makes little difference. In other words, the more intensely supervised facility-based programs i.e., inpatient or clinic-based programs, do not provide superior recovery to that seen in domiciliary or other forms of home program after unilateral TKA [30–40] or THA [39, 41–45] for uncomplicated patients. The randomized trial evidence is corroborated by several prospective and retrospective observational studies [12, 13, 23, 46–49]. We also know that the more intensely supervised facility-based programs including inpatient rehabilitation are delivered at far greater cost than programs not including inpatient rehabilitation [12, 13, 45] and may not be cost-effective [39, 50].

Despite the evidence base and despite considerable resources being used to identify, develop and assess recommendations or models of care at the state and national level in Australia [51–53], there is not yet a clear, unified evidence-based clinical practice guideline for rehabilitation following TKA or THA. The Royal Australasian College of Surgeons recently published a report recommending home-based rehabilitation services for appropriate patients, with the caveat that this would require structural changes to the care pathway [51]. In addition, models of care released by individual states identify home-based rehabilitation as the best option for the majority of people undergoing arthroplasty [52, 53]. The Australian Orthopaedic Association has identified the lack of clear, evidence-based clinical guidelines as a roadblock to successful TKA rehabilitation [54].

The absence of an evidence-based guideline can result in widespread practice variation, with many people not receiving the most appropriate care and limited healthcare resources being used sub-optimally. If the rehabilitation pathway provided following hip or knee arthroplasty was aligned with current evidence, patients would participate predominantly in a home-based therapy program, with intensely supervised facility-based programs reserved for people who require more assistance and support throughout the rehabilitation process [51, 55]. Consensus-based recommendations have been developed in North America [21]; no published data are available attesting whether these recommendations have influenced service delivery.

The primary aim of this study was to describe the most commonly utilized rehabilitation settings (facility- or home-based) by people who underwent elective TKA or THA secondary to osteoarthritis (OA). The secondary aims included to assess whether rehabilitation setting differed according to insurance status (public or private).

## Methods

### Design, hospital enrolment and ethical approval

This study was a planned sub-study of a large multi-centre, prospective observational cohort of people with knee or hip OA who underwent primary TKA or THA in 19 public and private Australian hospitals between August 2013 and January 2015. The study was prospectively registered in https://clinicaltrials.gov/ct2/show/NCT01899443 (July 2013).

Results of the larger study and other sub-studies have been reported previously [12, 13, 56–58] Initially, patients were enrolled using purposive sampling from a random selection of high-volume hospitals (performing over 275 knee or hip arthroplasties in 2012) in Australia. Later, enrolment included convenience sampling necessitated by the protracted time taken to gain departmental or executive approval using random sampling alone. The study was approved by multiple ethics committees (see Declarations for details), and written, informed consent was obtained from all participants.

### Patient screening and recruitment

Trained site coordinators screened and recruited study participants during their routine pre-operative assessment, typically within 6 weeks of surgery. The larger cohort included adults (age ≥ 18 years) with a primary diagnosis of OA in the index joint, with willingness and capacity to be followed-up by telephone post-surgery, and capacity to comprehend the study protocol, presented in English. People were excluded from the larger cohort if they were undergoing revision, hemi-arthroplasty or resurfacing surgery or had surgery planned on another joint within 3 months of the first. For the current study, we further restricted entry to people who would be eligible to undergo rehabilitation in any of the settings under consideration (home-based, facility-based or both), and therefore we excluded people who experienced a significant complication during the acute-care phase (Additional file 1), people undergoing bilateral surgeries, and self-funded participants as their insurance status changed across time; that is, for surgery they were typically coded as ‘privately insured’, but for their rehabilitation, they changed to being a ‘publicly insured’ patient. A subset of people from the larger cohort who were concurrently enrolled in a randomized rehabilitation trial at one of the participating sites were also excluded.

### Data and collection methods

At the pre-admission assessment, consenting participants provided socio-demographic and anthropometric details on a study proforma. Co-morbid conditions were recorded together with any prescription medication taken daily for any stated condition. Study participants also completed patient-reported outcome measures (the Oxford Knee or Hip Score (OKS or OHS) [12, 13] and the EuroQol ‘today’ health score (EQ. 0–100 scale) [12, 13]. The OKS and OHS each present 12 Likert-style questions pertaining to index joint pain and function. Each question is scored 0–4 based on degree of symptoms or impairment; the maximal score possible is 48, with higher scores indicating better status. Similarly, for the EQ scale, higher scores indicate better perceived health status.

Site coordinators recorded acute-care outcomes, including discharge destination, reasons for referral to inpatient rehabilitation (‘patient choice’, ‘surgeon choice’, ‘lack of social support’, ‘poor progress’, ‘post-operative complication’, ‘other’), and complications on a study proforma at the time of patient discharge.

Post-discharge outcomes were collected via telephone follow-up at 35 and 90 days by trained research officers (RO). To assist recall, a study diary was provided to participants at the time of enrolment. Outcomes of interest included length of stay (LOS) in an inpatient rehabilitation unit (where applicable), number of visits to physiotherapy clinics or day hospitals or use of domiciliary services, whether supervised physiotherapy was ongoing at 90 days or whether only an unmonitored home program (defined below) was followed since discharge from acute-care. The ROs confirmed details of rehabilitation ascertained from the site, the medical record audit and/or the previous telephone call as part of the follow-up process. We considered participants lost to follow-up if their 90-day assessment was not completed within 2 weeks of the due date or after a minimum five attempts to contact them.

### Data management

For quality control, study investigators re-abstracted medical record data provided by the site coordinator. Study personnel double-entered baseline, complication and discharge destination data, and corrected any disparities using data from the original patient study file.

### Primary outcome

The primary outcome, rehabilitation setting, was divided into two broad categories (facility-based or home-based) that we further split into five categories as follows:

Facility-based:

i) Rehabilitation in an inpatient unit *exclusively* (any number of days), following discharge from acute care (‘Inpatient only’)
ii) Rehabilitation in an inpatient unit (any number of days) followed by outpatient therapy including public or private physiotherapy clinic visits, or day-hospital visits; (‘Inpatient and outpatient’)
iii) Four or more treatments in an outpatient setting *exclusively,* including public or private physiotherapy clinic visits or day-hospital visits (‘Outpatient’)

Home-based rehabilitation:

iv) participation in a home-based program with no monitoring or supervision at any time, following discharge from acute care (‘Unmonitored home program’)
v) participation in a home-based program which included telerehabilitation or receipt of domiciliary visits up to the 90-day assessment, or attendance of up to three clinic-based (outpatient) sessions, (‘Monitored home program’). This threshold (up to three clinic sessions) was guided by published studies reporting the use of home programs following TKA or THA [30, 31, 37, 38, 42].

We also monitored participants’ use of community support packages, i.e., privately or publicly funded access to a limited number of services e.g. transport, meal provision, house cleaning, after discharge from the acute hospital as differences in access to such packages may explain differential reliance on inpatient rehabilitation between public and private sectors.

### Sample size and analyses

As this was a subset of participants from a larger study, the sample size was dictated by the sample size of the larger study and then by the number of participants who fulfilled the secondary eligibility criteria.

Following assessment of distributional assumptions, we used descriptive statistics as appropriate to describe the cohort. We compared characteristics of the study cohort and rehabilitation setting by insurance status (public or private) using unpaired, two-sided t-tests or Wilcoxon rank-sum tests for continuous variables and χ^2^ tests or Fisher’s exact tests, as appropriate. The knee and hip cohorts were analysed separately.

We completed all analyses using Stata Version 15.1 (www.stata.com; StataCorp., College Station, TX), and used *p* < 0.05 as the cut-off for statistical significance. We followed the STROBE guidelines for reporting.

## Results

### Study participants

**Fig. 1:**
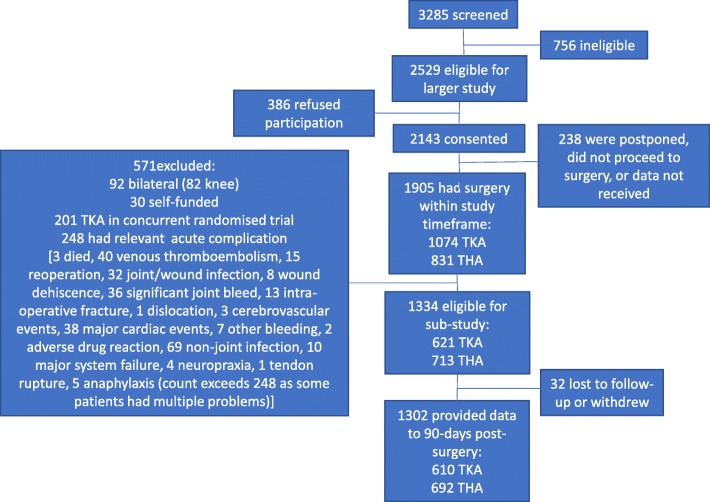
Cohort ascertainment and retention. TKA – total knee arthroplasty; THA – total hip arthroplasty

For the larger study, 1905 had surgery within the study timeframe from one of 19 hospitals (*n* = 9 private) (TKA *n* = 1074 [56%]; THA *n* = 831 [44%]); 1334 people (*n* = 621 TKA) were eligible for the sub-study after applying the additional exclusion criteria for the current study. We obtained complete data on rehabilitation to 90 days post-surgery on 1302 people (*n* = 610 TKA, *n* = 692 THA) (97%) (Fig. 1). Of the final cohort, 814 (62.5%) were privately insured, and the majority of these (*n* = 797, 97.9%), were operated upon in the private sector. All publicly insured participants were operated upon in the public sector.

Baseline characteristics of the cohort are shown in Table 1. Regardless of joint (hip or knee), compared to privately-insured participants, publicly-insured participants had a significantly higher BMI and worse pre-operative joint impairment (Oxford scores). Additionally, the proportion with a specific medical condition was often significantly greater amongst the publicly insured participants. Publicly-insured participants had a shorter acute hospital LOS.

**Table 1:** Cohort characteristics

Characteristic	Knee *N* = 610			Hip *N* = 692		
	Public *N* = 240 (%)	Private *N* = 370 (%)	*P*-value	Public *N* = 248 (%)	Private *N* = 444 (%)	*P*-value
Age mean (SD)	69.1 (8.6)	68.4 (8.4)	0.30	65.6 (10.5)	65.4 (10.4)	0.85
Female	106 (44.2)	159 (43)	0.77	129 (52)	203 (45.7)	0.11
Body mass index, mean (SD)	32.2 (6)	30.7 (5.7)	0.002	30.7 (6.6)	28.2 (4.9)	<.001
Body mass index category						
Underweight (< 18.5)	0	0	0.015	3 (1.2)	1 (0.2)	< 0.001
Normal (18.5–24.9)	21 (8.8)	47 (12.7)		46 (18.5)	116 (26.1)	
Obesity Class 1 (30–34.9)	68 (28.3)	118 (31.9)		58 (23.4)	94 (21.2)	
Obesity Class 2 (35–39.9)	51 (21.3)	45 (12.2)		36 (14.5)	26 (5.9)	
Extreme Obesity Class 3 (≥40)	21 (8.8)	23 (6.2)		24 (9.7)	13 (2.9)	
ASA^a^ score						
1	18 (7.5)	39 (10.5)	0.12	21 (8.5)	72 (16.2)	0.004
2	129 (53.8)	187 (50.5)		150 (60.5)	276 (62.2)	
3	73 (30.4)	132 (35.7)		69 (27.8)	88 (19.8)	
4	5 (2.1)	2 (0.5)		1 (0.4)	7 (1.6)	
Stroke	15 (6.3)	20 (5.4)	0.66	21 (8.5)	24 (5.4)	0.12
Hypertension	183 (76.3)	231 (62.4)	< 0.001	141 (56.9)	212 (47.7)	0.022
Heart disease	72 (30)	113 (30.5)	0.89	47 (19)	88 (19.8)	0.78
Kidney disease	13 (5.4)	11 (3)	0.13	10 (4)	6 (1.4)	0.024
Liver disease	6 (2.5)	13 (3.5)	0.48	8 (3.2)	6 (1.4)	0.093
Diabetes	59 (24.6)	54 (14.6)	0.002	19 (7.7)	47 (10.6)	0.21
Neurological disease	8 (3.3)	8 (2.2)	0.38	5 (2)	10 (2.3)	0.84
Respiratory disease	54 (22.5)	58 (15.7)	0.033	59 (23.8)	55 (12.4)	< 0.001
Current cancer	7 (2.9)	10 (2.7)	0.88	9 (3.6)	3 (0.7)	0.004
Diagnosed mental health condition	55 (22.9)	50 (13.5)	0.003	42 (16.9)	48 (10.8)	0.022
Sleep apnoea	21 (8.8)	31 (8.4)	0.87	21 (8.5)	13 (2.9)	0.001
Previous knee or hip arthroplasty	69 (28.8)	113 (30.5)	0.64	65 (26.2)	129 (29.1)	0.42
Other low back pain/lower limb pain^c^	113 (47.1)	172 (46.5)	0.89	140 (56.5)	211 (47.5)	0.024
Oxford Hip Score, mean (SD)	n/a	n/a		17 (7.9)	23.6 (8.9)	< 0.001
Oxford Knee Score, mean (SD)	21 (7.8)	24.1 (8.1)	<.001	n/a	n/a	
EuroQol today score, median (IQR)	75 (60, 85)	75 (65, 85)	0.76	75 (50, 85)	75 (60, 85)	0.062
LOS^b^ (days) median (IQR)	4 (4, 6)	6 (5, 7)	<.001	4 (3, 6)	5 (4, 6)	< 0.001

Values are N (%) unless specified otherwise

^a^American Society of Anesthesiologists physical status classification

^b^Length of stay in the acute hospital

^c^Affecting mobility

### Rehabilitation following TKA

Following TKA, 158 (25.9%) participated predominantly in home-based rehabilitation with greater participation observed amongst those publicly insured (32.9 vs 21.4%, *P* < 0.001) (Table 2). Referral to facility-based rehabilitation (inpatient rehabilitation and/or outpatient therapy) varied widely amongst hospitals. [Additional file 2: Figures S1 and S2, illustrate the variation in utilisation of the different rehabilitation settings at hospital level for both TKA and THA surgeries.] Referral to inpatient rehabilitation was more common amongst privately insured participants (56.0 (207/370) vs 7.5% (18/240), *P* < 0.001). Reasons for referral to inpatient rehabilitation varied by insurance status, with the predominant reason for referral for publicly insured participants being poor progress (8/18) whilst surgeon or patient choice (*n* = 177, [85.5%]) was the most common reason for privately insured participants (Additional file 2: Table S1). The difference in median LOS in inpatient rehabilitation between public and private participants did not reach statistical significance [Public 14 (10,21) vs Private 12 (8,14) days, *P* = 0.06].

**Table 2:** Setting of rehabilitation (home-based versus facility-based) by joint and insurance status

	Knee *N* = 610			Hip *N* = 692		
	Public *N* = 240 (%)	Private *N* = 370 (%)	*P*-value	Public *N* = 248 (%)	Private *N* = 444 (%)	*P*-value
Rehabilitation setting						
Home	79 (32.9)	79 (21.4)	0.001	176 (71)	247 (55.6)	< 0.001
Facility	161 (67.1)	291 (78.6)		72 (29)	197 (44.4)	
Home in detail			< 0.001			< 0.001
Unmonitored home	17 (7.1)	50 (13.5)		67 (27)	184 (41.4)	
Monitored home	46 (19.2)	18 (4.9)		95 (38.3)	33 (7.4)	
Domiciliary	16 (6.7)	11 (3)		14 (5.6)	30 (6.8)	
Facility in detail						
Outpatient only	143 (59.6)	84 (22.7)		61 (24.6)	50 (11.3)	
Inpatient only	1 (0.4)	26 (7)		3 (1.2)	40 (9)	
Inpatient and outpatient	17 (7.1)	181 (48.9)		8 (3.2)	107 (24.1)	

Privately-insured participants who received outpatient therapy (outpatient only or inpatient followed by outpatient) had a greater number of sessions (private: median [IQR] 8 (6,12) sessions; public: 6 [5, 8]; *P* < 0.001) (Table 3). Privately insured participants who participated in a home program also received more domiciliary visits (median [IQR] 9 (4,10) vs 4 (3,6), *P* = 0.019). Additional file 2: Table S2, summarises the types of outpatient-based rehabilitation received; variation in ‘type’ of intervention was evident within and between sectors. Telerehabilitation was not reported by any participant.

**Table 3:** Number of rehabilitation sessions by joint, rehabilitation setting and insurance status

Setting	Knee			Hip		
	Public	Private	*P*-value	Public	Private	*P*-value
Home, median (IQR)						
Unmonitored home	0	0		0	0	
Monitored home	2 (2, 3)	2 (1, 3)	0.13	2 (1, 3)	2 (1, 3)	0.64
Domiciliary	4 (3, 6)	9 (4, 10)	0.019	4.5 (3, 8)	5.5 (3, 8)	0.99
Facility, median (IQR)						
Outpatient +/− inpatient^a^	6 (5, 8)	8 (6, 12)	< 0.001	6 (4, 8)	8 (5, 11)	< 0.001

^a^Participants who received inpatient rehabilitation *only* are excluded from this table as no outpatient sessions were undertaken

At 90 days post-surgery, only 5% (12/240) of publicly-insured and 5.7% (21/370) of privately-insured patients were still receiving facility-based therapy (*P* = 0.72), and utilisation of community support packages was similar regardless of insurance status [Public 12.1% (29/240) vs Private 10% (37/370), *P* = 0.42].

### Rehabilitation received following THA

Following THA, 423 participants (61.1%) participated predominantly in a home-based program with greater participation observed amongst those publicly insured (71.0 vs 55.6%, *P* < 0.001) (Table 2). As for TKA, inpatient rehabilitation was more common amongst privately insured participants (33.1 vs 4.4%, *P* < 0.001). The predominant reason for referral to inpatient rehabilitation for publicly insured participants was poor progress (5%), whilst surgeon or patient choice (71.4%) was the most common reason for privately insured participants (Additional file 2: Table S2). Median LOS in inpatient rehabilitation was longer for publicly insured participants (14 [7, 28] vs 10 [7, 14] days; *P* = 0.022).

Privately-insured participants who received outpatient therapy (outpatient only or inpatient followed by outpatient) had a greater number of sessions (private: median [IQR] 8 [5, 11] sessions; public: 6 [4, 8]; *p* < 0.001) (Table 3). As seen with TKA participants, variation in the type of outpatient therapy following THA was evident within the sector and as well as between sectors (Additional file 2: Table S2).

A minority of THA recipients were still receiving facility-based therapy at 90 days post-surgery; the proportion was higher for privately insured recipients (Public 1.2% (3/248) vs Private 4.5% (20/444), *P* = 0.020). Utilisation of community support packages was similar regardless of insurance status [Public 12.1% (30/248) vs Private 11.7% (52/444), *P* = 0.88].

## Discussion

In this nationally-representative cohort we found that only 26% of TKA and 61% THA patients received predominantly home-based rehabilitation. Greater facility-based utilisation was evident for privately insured patients, both in terms of rate of utilisation and number of treatment sessions. We also observed wide variation in the utilisation of inpatient rehabilitation and in the forms of outpatient-based programs (e.g. group, one-to-one), both within and between insurance sectors, regardless of the surgery type. These observations are more than academic; facility-based programs are provided at greater cost to the payer, whether it is a patient, a government, or a private insurer. Inpatient rehabilitation programs in particular add many thousands of dollars to the episode-of-care costs [12, 13, 39, 45, 50], and have been shown to subject patients to potentially unnecessary diagnostic interventions [59], and may be associated with more adverse events [60, 61], hence, from the perspective of the payer, it is important to both question and monitor the use of the more intensely supervised facility-based programs.

Our findings concur with a recent retrospective survey of arthroplasty recipients conducted in the Netherlands that concluded that facility-based therapy remained the norm after arthroplasty [24], and one-to-one outpatient-based therapy was very common. In contrast to the latter study, however, where it was observed that 47% of recipients continue supervised therapy beyond 3-months post-surgery, we observed few recipients continuing facility-based therapy at the 3-month (90-day) point. Similarly, our variation in hospital-level utilisation of different settings support the observations of a recent very large retrospective study (*n* > 18,000) conducted in the Unites States revealing vast geographic variation in rehabilitation settings following TKA as well as variation in length of therapy episode ranging from an average of 33 (95% CI 28–41) days though to 42 (36–50) days [62].

The variation we observed in the utilization of inpatient rehabilitation corroborates the variation reported by Australia’s largest private healthcare insurer - that private hospital-level referral to inpatient rehabilitation varies from 0 to 100% [63]. Similarly, just as our data found that referral for private patients was not predominantly based on need (but rather predominantly patient or surgeon choice (preference)), the aforementioned study reported that patient factors such as age, gender, comorbidity index and even hospital-acquired complication explained little of the variation in referral rates between hospitals [63]. Despite having less pre-operative impairment, obesity and comorbidity than publicly insured patients, in the current study, privately insured patients reported higher utilisation of facility-based rehabilitation, suggesting that factors other than need predominantly drive what rehabilitation is ultimately received. Equally surprising, publicly insured recipients had a shorter acute-care LOS despite their higher level of impairment pre-operatively. If inpatient rehabilitation is underutilised in the public sector due to lack of availability, we might expect to see an extended acute-care LOS, especially given the publicly insured patients’ greater burden of comorbidity. Further, we may expect to see far greater use of community packages by the public sector in order to compensate for limited availability of inpatient rehabilitation beds, but utilisation rates across the sectors were similar.

As the number of arthroplasty surgeries grows, along with the associated costs, the alignment of rehabilitation setting with the evidence – particularly if led by a shift away from inpatient rehabilitation programs - offers an opportunity to improve the efficiency of TKA and THA surgeries. With this goal in mind, the development of evidence-based guidelines would be a reasonable place to start though we acknowledge that they alone will not likely remove the evidence-practice gap. Changes to reimbursement models between provider and insurer, and consumer and clinician education around the efficacy of the programs available so that evidence rather than preference predominantly drives what is received, will also be required. We note that guidelines are being developed in the United Kingdom [64], but country-specific guidelines are likely required owing to the different drivers of care that prevail in different healthcare systems. Further, we necessarily excluded people with major acute complications and those undergoing bilateral surgeries. Such persons may benefit more from the more intensively supervised facility-based therapies, thus, a priority area for future research is the determination of who benefits most from the more intensely supervised programs.

### Strengths and limitations

To the best of our knowledge, this is the first large-scale, prospective review of what rehabilitation settings are commonly used by TKA and THA recipients. We report rehabilitation setting used across a large, clearly defined homogeneous cohort, followed prospectively with deliberate exclusion of those who were not equally eligible for home-based and facility-based rehabilitation. The characteristics of the cohort reflect those reported in previous Australian studies [16, 30, 34, 35, 38, 41, 42, 44, 46]. The higher proportion of private recipients in this cohort (62.5%) reflects that of the total arthroplasty cohort in Australia, as do our age and BMI profiles [7], thus, the patterns observed should be broadly generalisable to the greater Australian arthroplasty population. That our rates of inpatient rehabilitation utilisation amongst private hospitals align with those reported by Australia’s largest insurer support the generalisability of our data. Our observations are relevant for healthcare sectors elsewhere - such as the United States [47, 48, 50, 63, 65] and Germany [66] - where sizeable private sectors provide arthroplasty services to those privately insured. Our observations and discussion are also relevant to health systems that already rely predominantly on outpatient- and home-based programs such as the United Kingdom [25], Canada [21], and the Netherlands [24, 66] because they also highlight the variation that persists in community-based programs and between what is provided for knee and hip arthroplasty recipients, and bring to the fore the uncertainty about the appropriate duration of community-based rehabilitation following either surgery.

A limitation of the study is the reliance on patient recall to describe rehabilitation received. Whilst we incorporated several mechanisms to reduce recall bias including questioning of rehabilitation received at both 35 and 90 days post-surgery, use of patient recall diaries, and hospital-level evidence of inpatient rehabilitation admission, it is possible that utilisation of outpatient-based therapy was mis-reported. Further, we provide a snapshot of rehabilitation received (setting and number of treatments) by individuals without regard for individual needs. Whilst we acknowledge there may be some patients who require more supervised therapy – and we excluded those for whom this may be most obvious - we believe that it is unlikely that variation in individual need explains much of the variation observed. This is most apparent when we consider the lower rates of participation in facility-based therapy (both in terms of proportion of recipients and number of treatment sessions) amongst the publicly insured recipients despite their greater pre-operative joint impairment, BMI and comorbid burden, and shorter acute hospital length of stay. Our utilisation rates may not be generalisable to remote locations; no participant hospital was located in a remote region and this may explain why no participant reported the use of telerehabilitation. Finally, as the focus of this paper is on the utilisation of different rehabilitation settings, we have purposely not reported outcomes including satisfaction with the program received. We have previously reported outcomes for this cohort between those who did and did not have inpatient rehabilitation and found no differences in patient-reported joint-specific and health-related quality of life scores at 35, 90 and 365 days post-surgery [12, 13]. We acknowledge, however, that participant satisfaction with particular rehabilitation settings and programs may be contributing to the evidence-practice gap we have observed.

## Conclusion

Contrary to the body of evidence derived from randomised trials and prospective observational studies indicating home-based programs yield similar recovery profiles, the more intensely supervised facility-based rehabilitation remains the norm for most TKA and many THA recipients, and more so for those privately insured despite presenting with less joint-related impairment and obesity prior to surgery than those publicly insured.

These observations suggest that current practice in Australia is not primarily guided by the available evidence or need and is likely provided at a far greater cost than it would be if the best available evidence were adopted. Resolving this evidence-practice gap should be a priority for all stakeholders. The development of evidence-based guidelines in this area is needed.

## Additional files

Additional file 1: Acute complications that rendered a patient ineligible for the sub-study. List of complications. (PDF 16 kb)

Additional file 2: Additional detail about rehabilitation setting. **Figure S1.** Variation in rehabilitation setting by hospital: TKA. **Figure S2**. Variation in rehabilitation setting by hospital: THA. **Table S1.** Reasons for referral to inpatient rehabilitation as provided by the hospital. **Table S2.** Type of facility-based outpatient rehabilitation sessionsa by insurance status. (PDF 6967 kb)

## Abbreviations

ASA: American Society of Anesthesiologists
CI: Confidence interval
IQR: Interquartile range
LOS: Length of stay
OA: Osteoarthritis
OHS: Oxford Hip Score
OKS: Oxford Knee Score
RO: Research officer
THA: Total hip arthroplasty
TKA: Total knee arthroplasty
χ^2^: Chi square

## References

[1] Ackerman IN, Bohensky MA, de Steiger R (2017). Substantial rise in the lifetime risk of primary total knee replacement surgery for osteoarthritis from 2003 to 2013: an international, population-level analysis. Osteoarthr Cartil.

[2] Ackerman IN, Bohensky MA, de Steiger R (2017). Lifetime risk of primary total hip replacement surgery for osteoarthritis from 2003 to 2013: a multinational analysis using National Registry Data. Arth Care Res.

[3] Ferket BS, Feldman Z, Zhou J (2017). Impact of total knee replacement practice: cost effectiveness analysis of data from the osteoarthritis initiative. BMJ.

[4] Badley EM, Canizares M, Perruccio AV (2017). Population-based study of changes in arthritis prevalence and arthritis risk factors over time: generational differences and the role of obesity. Arthritis Care Res (Hoboken).

[5] Carr AJ, Robertsson O, Graves S (2012). Knee replacement. Lancet..

[6] Dowsey MM, Liew D, Choong PF (2011). Economic burden of obesity in primary total knee arthroplasty. Arthritis Care Res (Hoboken).

[7] National Joint Replacement Registry. Demographics of hip, knee and shoulder arthroplasty. Supplementary report 2018. AOANJRR.

[8] Nilsdotter AK, Toksvig-Larsen S, Roos EM (2009). A 5 year prospective study of patient-relevant outcomes after total knee replacement. Osteoarthr Cartil.

[9] Bengtsson A, Donahue GS, Nemes S, Garellick G, Rolfson O (2017). Consistency in patient-reported outcomes after total hip replacement a 6-year registry follow-up of 15,755 patients. Acta Orthop.

[10] Arden NK, Kiran A, Biant LC (2011). What is a good patient reported outcome after total hip replacement?. Osteoarthr Cartil.

[11] March LM, Cross MJ, Lapsley H (1999). Outcomes after hip or knee replacement surgery for osteoarthritis. Med J Aust.

[12] Naylor JM, Hart A, Mittal R, Harris IA, Xuan W (2018). The effectiveness of inpatient rehabilitation after uncomplicated total hip arthroplasty: a propensity score matched cohort. BMC Musculoskelet Disord.

[13] Naylor JM, Hart A, Mittal R, Harris IA, Xuan W (2017). The effectiveness of inpatient rehabilitation after uncomplicated total knee arthroplasty: a propensity score matched cohort. Med J Aust.

[14] Meier W, Mizner RL, Marcus RL (2008). Total knee arthroplasty: muscle impairments, functional limitations, and recommended rehabilitation approaches. J Orthop Sports Phys Ther.

[15] Noble PC, Gordon MJ, Weiss JM, et al. Does total knee replacement restore normal knee function? Clin Orthop Relat Res. 2005;(431):157–65. 10.1097/01.blo.0000150130.03519.fb 15685070

[16] Ko V, Naylor JM, Harris IA (2013). The six-minute walk test is an excellent predictor of functional ambulation after total knee arthroplasty. BMC Musculoskelet Disord.

[17] Stevens-Lapsley JE, Balter JE, Kohrt WM, Eckhoff DG (2010). Quadriceps and hamstrings muscle dysfunction after total knee arthroplasty. Clin Orthop Relat Res.

[18] Hudd DL, Dennis DA, Thomas AC, Wolfe P, Dayton MR, Stevens-Lapsley JE (2014). Muscle strength and functional recovery during the first year after THA. Clin Orthop Relat Res.

[19] Fukumoto Y, Ohata K, Tsukagoshi R (2013). Changes in hip and knee muscle strength in patients following total hip arthroplasty. J Jpn Phys Ther Assoc.

[20] Naylor JM, Harmer AR, Walker R, Partridge C (2007). Physiotherapy rehabilitation following TKR. Recent advances in physiotherapy.

[21] Westby MD, Brittain A, Backman CL (2014). Expert consensus on best practices for post-acute rehabilitation after total hip and knee arthroplasty: a Canada and United States Delphi study. Arthritis Care Res.

[22] Peter WF, Nelissen RG, Vlieland TP (2014). Guideline recommendations for post-acute postoperative physiotherapy in total hip and knee arthroplasty: are they used in daily clinical practice?. Musculoskeletal Care.

[23] Benz T, Angst F, Oesch P (2015). Comparison of patients in three different rehabilitation settings after knee or hip arthroplasty: a natural observational, prospective study. BMC Musculoskelet Disord.

[24] Peter WF, Tilbury C, Verdegaal SHM (2016). The provision of preoperative and postoperative physical therapy in elderly people with hip and knee osteoarthritis undergoing primary joint replacement surgery. Current Orthopaedic Practice.

[25] Okoro T, Ramavath A, Howarth J (2013). What does standard rehabilitation practice after total hip replacement in the UK entail? Results of a mixed methods study. BMC Musculoskelet Disord.

[26] Artz N, Dixon S, Wylde V, Beswick A, Blom A, Gooberman-Hill R (2013). Physiotherapy provision following discharge after total hip and total knee replacement: a survey of current practice at high-volume NHS hospitals in England and Wales. Musculoskeletal Care.

[27] Naylor J, Harmer A, Fransen M, Crosbie J, Innes L (2006). Status of physiotherapy rehabilitation after total knee replacement in Australia. Physiother Res Internat.

[28] Artz N, Elvers KT, Lowe CM (2015). Effectiveness of physiotherapy exercise following total knee replacement: systematic review and meta-analysis. BMC Musculoskelet Disord.

[29] Buhagiar M, Naylor JM, Harris IA, Xuan W, Adie S, Lewin A JAMA Netw Open 2019;2(4):e192810. 10.1001/jamanetworkopen.2019.281 10.1001/jamanetworkopen.2019.2810 PMC6487570 31026026

[30] Ko V, Naylor J, Harris I, Crosbie J, Yeo A, Mittal R (2013). One-to-one therapy is not superior to group or home-based therapy after total knee arthroplasty: a randomized, superiority trial. J Bone Joint Surg A.

[31] Kramer JF, Speechley M, Bourne M, Rorabeck C (2003). Comparison of clinic- and home-based rehabilitation programs after total knee arthroplasty. Clin Orthop Relat Res.

[32] Rajan RA, Pack Y, Jackson H, Gillies C, Asirvatham R (2004). No need for outpatient physiotherapy following total knee arthroplasty. Acta Orthop Scand.

[33] Mockford BJ, Thompson NW, Humphreys P, Beverland DE (2008). Does a standard outpatient physiotherapy regime improve the range of knee motion after primary total knee arthroplasty?. J Arthroplast.

[34] Han ASY, Nairn L, Harmer AR, Crosbie J, March L, Parker D (2015). A multicenter non-inferiority randomized clinical trial comparing a home exercise program with usual outpatient care. Arthritis Car Res.

[35] Russell TG, Buttrum P, Wootton R, Jull GA (2011). Internet-based outpatient telerehabilitation for patients following total knee arthroplasty: a randomized controlled trial. J Bone Joint Surg A.

[36] Tousignant M, Moffet H, Boissy P, Corriveau H, Cabana F, Marquis F (2011). A randomized controlled trial of home telerehabilitation for post-knee arthroplasty. J Telemed Telecare.

[37] Madsen M, Larsen K, Kirkegard Madsen I, Soe H, Hansen TB (2013). Late group-based rehabilitation has no advantages compared with supervised home-exercises after total knee arthroplasty. Dan Med J.

[38] Buhagiar M, Naylor JM, Harris IA, Xuan W, Kohler F, Wright R (2017). Effect of inpatient rehabilitation vs a monitored home-based program on mobility in patients with total knee arthroplasty. The HIHO randomized clinical trial. JAMA..

[39] Mahomed NN, Davis AM, Hawker G, Badley E, Davey JR, Syed KA (2008). Inpatient compared with home-based rehabilitation following primary unilateral total hip or knee replacement: a randomized controlled trial. J Bone Joint Surg Am.

[40] Fleischman AN, Crizer MP, Tarabichi M, Smith S, Rothman RH, Lonner JH, Chen AF (2019). Recovery of knee flexion with unsupervised home exercise is not inferior to outpatient physical therapy after TKA: a randomized trial. Clin Orthop Relat Res.

[41] Coulter CL, Scarvell JM, Neeman TM, Smith PN (2013). Physiotherapist directed rehabilitation exercises in the outpatient or home setting improve strength, gait speed, and cadence after elective total hip replacement: a systematic review. J Physiother.

[42] Galea MP, Levinger P, Lythgo N, Cimoli C, Weller R, Tully E (2008). A targeted home- and center-based exercise program for people after total hip replacement: a randomized clinical trial. Arch Phys Med Rehabil.

[43] Austin MS, Urbani BT, Fleischman AN, Fernando ND, Purtill JJ, Hozack WJ (2017). Formal physical therapy after total hip arthroplasty is not required: a randomized controlled trial. J Bone Joint Surg Am.

[44] Coulter C, Perriman DM, Neeman TM, Smith PN, Scarvell JM (2017). Supervised or unsupervised rehabilitation after total hip replacement provides similar improvements for patients: a randomised controlled trial. Arch Phys Med Rehabil.

[45] Mikkelsen LR, Mechlenburg I, Søballe K, Jørgensen LB, Mikkelsen S, Bandholm T (2014). Effect of early supervised progressive resistance training compared to unsupervised home-based exercise after fast-track total hip replacement applied to patients with preoperative functional limitations. A single-blinded randomised controlled trial. Osteoarthr Cartil.

[46] Tribe KL, Lapsley HM, Cross MJ (2005). Selection of patients for inpatient rehabilitation or direct home discharge following total joint replacement surgery: a comparison of health status and out-of-pocket expenditure of patients undergoing hip and knee arthroplasty for osteoarthritis. Chronic Illn.

[47] Mallinson TR, Batema J, Tseng H-Y, Manheim L, Almagor O, Deutsch A, Heinemann AW (2011). A comparison of discharge functional status after rehabilitation in skilled nursing, home health, and medical rehabilitation settings for patients after lower-extremity joint replacement surgery. Phys Med Rehabil.

[48] Padgett DE, Christ AB, Joseph AD, Lee Y-Y, Haas SB, Lyman S (2018). Discharge to inpatient rehab does not result in improved functional outcomes following primary total knee arthroplasty. J Arthroplast.

[49] Hutchinson AG, Gooden B, Lyons MC, Roe JP, O’Sullivan MD, Salmon LJ (2018). Inpatient rehabilitation did not positively affect 6-month patient-reported outcomes after hip or knee arthroplasty. ANZ J Surg.

[50] London DA, Vilensky S, O'Rourke C, Schill M, Woicehovich L, Froimson MI (2016). Discharge disposition after joint replacement and the potential for cost savings: effect of hospital policies and surgeons. J Arthroplast.

[51] Royal Australasian College of Surgeons 2018, Rehabilitation pathways following hip and knee arthroplasty, Royal Australasian College of surgeons, North Adelaide. Royal College of surgeons. https://www.surgeons.org/media/25621953/2018-01-29_mbp_arthroplasty_final.pdf Accessed Jan 2019.

[52] WA Department of Health, Western Australia (2010). Elective Joint Replacement Service Model of Care.

[53] Statewide Orthopaedic Clinical Network and Rehabilitation Clinical Network, South Australia. Models of Care for Orthopaedic Rehabilitation - Fragility Fractures, General Orthopaedic Trauma and Arthroplasty; 2011. https://www.sahealth.sa.gov.au/wps/wcm/connect/443d0f8046e616d78953fb2e504170d4/Models+of+Care-SSS-Clinical+Network-20110509.pdf?MOD=AJPERES&CACHEID=ROOTWORKSPACE-443d0f8046e616d78953fb2e504170d4-lmsFpP0 Accessed Jan 2019.

[54] Australian Orthopaedic Association Media Release. Knee rehabilitation needs clinical guidelines to be better understood. https://www.aoa.org.au/docs/default-source/advocacy/aoa-media-release_knee-rehabilitation-services.pdf ). Accessed Jan 2019.

[55] Naylor JM, Crosbie J, Ko V (2015). Is there a role for rehabilitation streaming following TKA? Preliminary insights from a randomised controlled trial. J Rehab Med.

[56] Chua M, Hart A, Harris IA, Mittal R, Xuan W, Naylor JM (2017). Early mobilisation after total hip or knee arthroplasty: a multicentre prospective observational study. PLoS One.

[57] Mayer M, Naylor J, Harris I, Badge H, Adie S, Mills K (2017). Evidence base and practice variation in acute care processes for knee and hip arthroplasty surgeries. PLoS One.

[58] Naylor JM, Descallar J, Grootemaat M, Badge H, Harris IA, Simpson G (2016). Is satisfaction with the acute-care experience higher amongst consumers treated in the private sector? A survey of public and private sector arthroplasty recipients. PLoS One.

[59] White PB, Carli AV, Meftah M, Ghazi N, Alexiades MM, Windsor RE (2018). Patients discharged to inpatient rehabilitation facilities undergo more diagnostic interventions with no improvement in outcomes. Orthopedics.

[60] Bini SA, Fithian DC, Paxton LW, Khatod MX, Inacio MC, Namba RS (2010). Does discharge disposition after primary total joint arthroplasty affect readmission rates?. J Arthroplast.

[61] McLawhorn AS, Fu MC, Schairer WW, Sculco PK, Maclean CH, Padgett DE (2017). Continued inpatient care after primary total knee arthroplasty increases 30-day post-discharge complications: a propensity score-adjusted analysis. J Arthroplast.

[62] Warren M, Shireman T (2018). Geographic variability in discharge setting and outpatient Postacute physical therapy after Total knee arthroplasty: a retrospective cohort study. Phys Ther.

[63] Schilling C, Keating C, Barker A, Wilson SF, Petrie D (2018). Predictors of inpatient rehabilitation after total knee replacement: an analysis of private hospital claims data. Med J Aust.

[64] NICE. National Institute for Health and Care Excellence. Joint replacement (primary): hip, knee and shoulder. https://www.nice.org.uk/guidance/indevelopment/gid-ng10084 Accessed Mar 2019. 32881469

[65] Hart A, Bergeron SG, Epure L, Huk O, Zukor D, Antoniou J (2015). Comparison of US and Canadian perioperative outcomes and hospital efficiency after total hip and knee arthroplasty. JAMA Surg.

[66] Seeber GH, Wijnen A, Lazovic D, Bulstra SK, Dietz G, Van Lingen CP (2017). Effectiveness of rehabilitation after a total hip arthroplasty: a protocol for an observational study for the comparison of usual care in the Netherlands versus Germany. BMJ Open.

