# Supplementary material for: Variation in rehabilitation setting after uncomplicated total knee or hip arthroplasty: a call for evidence-based guidelines
Source: BMC Musculoskelet Disord. 2019 May 15;20:214. doi: 10.1186/s12891-019-2570-8 (PMC6521339; doi:10.1186/s12891-019-2570-8)
Supplement: Supplementary file 1 — Acute complications that rendered a patient ineligible for the sub-study. List of complications. (PDF 16 kb) [file 12891_2019_2570_MOESM1_ESM.pdf]

Additional File 1

Variation in rehabilitation setting after uncomplicated total knee or hip arthroplasty: a call for evidence-based guidelines

Naylor JM, Hart A, Harris IA, Lewin A

Description of how complications were defined to determine who was excluded

Using the validated Clavien-Dindo (CD)[1] surgical complication classification system, we defined a significant complication as CD Grade 3 or above, i.e., requiring surgical management or being life threatening, e.g revision surgery or myocardial infarction. We also included in our definition of significant complications those complications or events that did not meet the CD Grade 3 criteria but were considered to potentially influence the rehabilitation pathway or participation in rehabilitation, e.g., deep vein thrombosis or motor neuropraxia.

References

1. Dindo D, Demartines N, Clavien PA. Classification of surgical complications: a new proposal with evaluation in a cohort of 6336 patients and results of a survey. *Ann Surg* 2004;240: 205-213.
