# Supplementary material for: Variation in rehabilitation setting after uncomplicated total knee or hip arthroplasty: a call for evidence-based guidelines
Source: BMC Musculoskelet Disord. 2019 May 15;20:214. doi: 10.1186/s12891-019-2570-8 (PMC6521339; doi:10.1186/s12891-019-2570-8)
Supplement: Supplementary file 2 — Additional detail about rehabilitation setting. Figure S1. Variation in rehabilitation setting by hospital: TKA. Figure S2. Variation in rehabilitation setting by hospital: THA. Table S1. Reasons for referral to inpatient rehabilitation as provided by the hospital. Table S2. Type of facility-based outpatient rehabilitation sessionsa by insurance status. (PDF 6967 kb) [file 12891_2019_2570_MOESM2_ESM.pdf]

Additional File 2

Variation in rehabilitation setting after uncomplicated total knee or hip arthroplasty: a call for evidence-based guidelines

Naylor JM, Hart A, Harris IA, Lewin A

Figure S1. Variation in rehabilitation setting by hospital: TKA

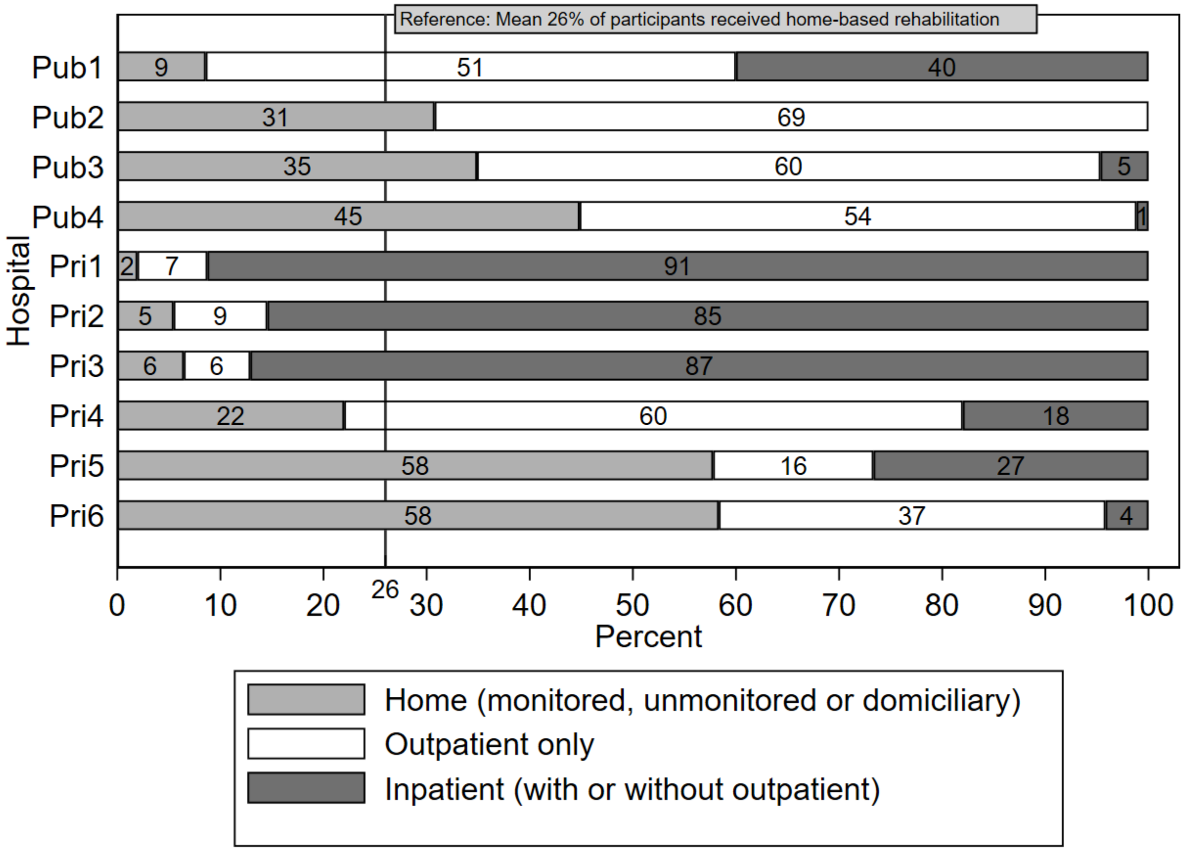

Legend: Only hospitals providing 20 or more patients included in the proportions; Pub = public; Pri = private.

Figure 2. Variation in rehabilitation setting by hospital: THA

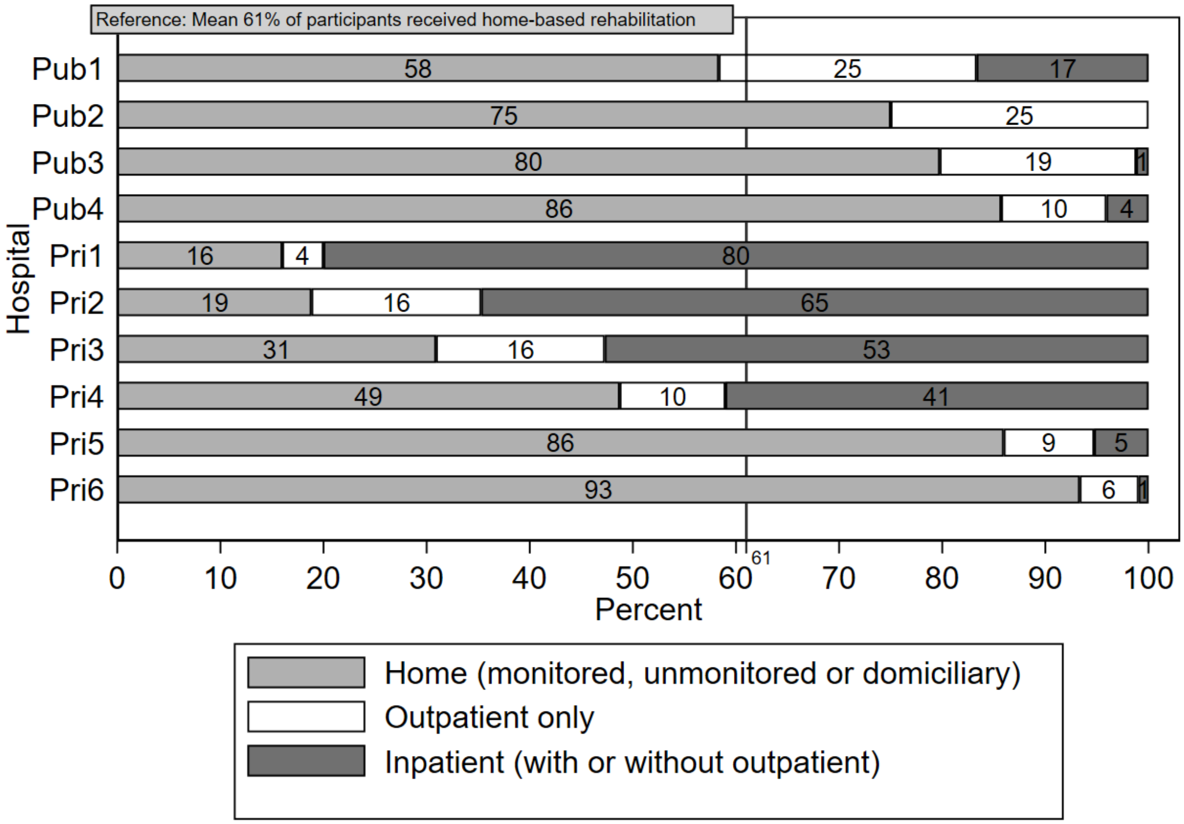

Legend: Only hospitals providing 20 or more patients included in the proportions; Pub = public; Pri = private

Table S1. Reasons for referral to inpatient rehabilitation as provided by the hospital

Reason	Knee, N (%)			Hip, N (%)		
	**Public N=18**	**Private N=207**	**P-value**	**Public N=11**	**Private N=147**	**P-value**
			< 0.001			< 0.001
Surgeon choice	1 (5.6)	119 (57.5)		1 (9.1)	71 (48.3)	
Patient choice	1 (5.6)	58 (28)		0	34 (23.1)	
Post-operative complication	1 (5.6)	0		2 (18.2)	0	
Poor progress	8 (44.4)	4 (1.9)		7 (63.6)	7 (4.8)	
Other	0	0		0	1 (0.7)	
Lack of social support	7 (38.9)	11 (5.3)		0	20 (13.6)	
Missing	0	15 (7.2)		1 (9.1)	14 (9.5)	

Three publicly insured patients were recorded by the hospital as being referred to inpatient rehabilitation due to a complication. These complications (pressure area on buttock; faecal incontinence; severe vomiting and anaemia) did not meet the criteria for a complication as per our definition.

Table S2. Type of facility-based outpatient rehabilitation sessions[a] by insurance status

Type	Knee, N (%)			Hip, N (%)		
	**Public N=160**	**Private N=265**	**P-value**	**Public N=69**	**Private N=157**	**P-value**
One-to-one	92 (57.5)	140 (52.8)	0.35	43 (62.3)	72 (45.9)	0.023
Gym	66 (41.3)	36 (13.6)	<.001	22 (31.9)	17 (10.8)	<0.001
Water (group or one-to-one)	20 (12.5)	31 (11.7)	0.81	13 (18.8)	33 (21)	0.71
Hospital Day program	9 (5.6)	110 (41.5)	<.001	2 (2.9)	78 (49.7)	<.001

[a]Restricted to people who were in the facility-based rehabilitation group; excludes gym or water-based sessions delivered as part of an inpatient program.
